# Progression of breast cancer following locoregional ipsilateral recurrence: importance of interval time

**DOI:** 10.1038/bjc.2016.135

**Published:** 2016-05-26

**Authors:** Jennifer C Melvin, Arnie D Purushotham, Hans Garmo, Sarah E Pinder, Ian S Fentiman, Cheryl Gillett, Anca Mera, Margreet Lüchtenborg, Lars Holmberg, Mieke Van Hemelrijck

**Correction to:** *British Journal of Cancer* (2016) **114**, 88–95; doi:10.1038/bjc.2015.314; published online 10 December 2015

Upon publication of the above paper in the *British Journal of Cancer*, one of the authors identified an error in the spelling of their name. Margreet Lüchtenborg appears correctly in the author list above.

